# Health-related quality of life, developmental milestones, and self-esteem in young adults with bleeding disorders

**DOI:** 10.1007/s11136-017-1696-0

**Published:** 2017-09-12

**Authors:** P. F. Limperg, L. Haverman, H. Maurice-Stam, M. Coppens, C. Valk, M. J. H. A. Kruip, J. Eikenboom, M. Peters, M. A. Grootenhuis

**Affiliations:** 1 Psychosocial Department, Emma Children’s Hospital, AMC, Postbox 22660, 1100 DD Amsterdam, The Netherlands; 2 Amsterdam Public Health Research Institute, Amsterdam, The Netherlands; 3 Department of Vascular Medicine, Hemophilia Comprehensive Care Treatment Center, AMC, Amsterdam, The Netherlands; 4 Department of Hematology, Hemophilia Comprehensive Care Treatment Center, Erasmus University Medical Center, Rotterdam, The Netherlands; 5 Department of Thrombosis and Hemostasis, Leiden University Medical Center, Leiden, The Netherlands; 6 Department of Pediatric-Hematology, Emma Children’s Hospital and Hemophilia Comprehensive Care Treatment Center, AMC, Amsterdam, The Netherlands

**Keywords:** Hemophilia, Young adults, Health-related quality of life, Developmental milestones, Self-esteem

## Abstract

**Background:**

The treatment of bleeding disorders improved in the last decades. However, the effect of growing up with bleeding disorders on developmental, emotional, and social aspects is understudied. Therefore, this study assesses HRQOL, developmental milestones, and self-esteem in Dutch young adults (YA) with bleeding disorders compared to peers.

**Methods:**

Ninety-five YA (18–30 years) with bleeding disorders (78 men; mean 24.7 years, SD 3.5) and 17 women (mean 25.1 years, SD 3.8) participated and completed the Pediatric Quality of Life Inventory Young Adult version, the Course of Life Questionnaire, and the Rosenberg Self-Esteem Scale. Differences between patients with bleeding disorders and their peers, and between hemophilia severity groups, were tested using Mann–Whitney *U* tests.

**Results:**

YA men with bleeding disorders report a slightly lower HRQOL on the total scale, physical functioning, and school/work functioning in comparison to healthy peers (small effect sizes). YA men with severe hemophilia report more problems on the physical functioning scale than non-severe hemophilia. YA men with bleeding disorders achieved more psychosexual developmental milestones than peers, but show a delay in ‘paid jobs, during middle and/or high school.’ A somewhat lower self-esteem was found in YA men with bleeding disorders in comparison to peers (small effect size). For YA women with bleeding disorders, no differences were found on any of the outcomes in comparison to peers.

**Conclusion:**

This study demonstrates some impairments in HRQOL and self-esteem in YA men with bleeding disorders. By monitoring HRQOL, problems can be identified early, especially with regard to their physical and professional/school functioning.

## Introduction

The term bleeding disorder refers to a deficiency of the blood clotting system, in which bleeding is prolonged and excessive. Hemophilia A and B and von Willebrand (VWD) are the most common bleeding disorders identified [1]. Hemophilia is X-linked; women are carriers and men are affected. Symptoms of hemophilia are spontaneous and posttraumatic bleeds [2]. Other inherited bleeding disorders, such as VWD, affect men and women. For those, mildly affected patients may suffer from frequent nosebleeds and bruises, while patients with a more severe type may have symptoms similar to those of hemophilia [1]. For women with bleeding disorders, heavy menstruation and postpartum bleeding are common [3].

Mid previous century people with bleeding disorders could only be treated with whole blood or fresh plasma obtained from donors [4]. Most people affected by severe hemophilia died in childhood or in early adulthood [4, 5]. Since the 1980s, as a result of the hepatitis and human immunodeficiency virus (HIV) crises that occurred in Western Europe and the US due to contaminated blood products, safe plasma concentrates of coagulation factors have been produced, and genetically engineered factors led to the industrial production of factor products [2, 4, 6]. Prophylactic treatment was used in order to prevent bleeding and joint damage, ultimately allowing patients to maintain a near normal lifestyle [7].

Despite the available adequate treatment, patients with bleeding disorders still endure difficulties and impairments in daily life, such as hospital visits, frequent injections, and limited participation in (sport) activities [8]. A vulnerable group of patients with bleeding disorders are adolescents and young adults (YA). Transition into adulthood is a critical phase for all children, but growing up with a bleeding disorder brings additional challenges compared with healthy children; acceptance of the limitations of the bleeding disorder, the shift from parental care to self-care, the move from a pediatric to an adult treatment facility, the search for employment that provides adequate medical insurance, or starting a family [9, 10]. Adolescents with bleeding disorders usually start with learning self-infusion around the age of eleven, a procedure which involves complex self-management skills [11, 12]. Despite the advantages of being able to provide care at home (e.g., less hospital visits, more independence), the large responsibility for management of the bleeding disorder is burdensome, especially for patients with hemophilia [13, 14].

During childhood, psychosocial care in the Netherlands is extensive for children with bleeding disorders [15], to prepare them for the transition period. Patients are expected to be independent and fully responsible for their own treatment when they are 18 years making the transition to adult healthcare challenging. Research in YA with hemophilia in Canada showed that YA have more joint disease and slightly worse HRQOL with regard to physical functioning and pain than their younger counterparts and healthy peers [16]. On top of that, research has demonstrated that the mean age of YA for complete self-management and responsibility is not reached until they are 22 years [11].

To identify what elements are relevant and impact daily life of YA (ages 18–30 years) with bleeding disorders, we conducted a foregoing qualitative study. Focus groups revealed that, despite growing up in this era and in a developed country with adequate treatment available, YA still do experience obstacles and issues in daily life with regard to their treatment and condition [17]. For example, being autonomous (in relation to parents, travel, sports, professionally), as well as development of self-esteem (illness perception, acceptance, and being able to pursue ambitions as much as healthy peers), were identified as major issues [17].

These qualitative results have led us to conceive the current study, in which we aim to quantify our findings in a larger group of YA with bleeding disorders. By administering validated questionnaires we can capture the themes retrieved from the focus groups, in a larger sample of YA with bleeding disorders. By comparing them to the peers from the general population, we hope to gain insight into the HRQOL, psychosocial development, and self-esteem of YA with bleeding disorders. Furthermore, in this study, we will examine the influence of severity of hemophilia on these outcomes in YA men with hemophilia, as this is the largest group.

## Methods

### Participants

Eligible patients were YA aged 18–30 years with hemophilia A/B [clotting factor <1% (severe) or 1–40% (non-severe)], VWD, or other congenital bleeding disorders. Patients were treated in one of the three participating treatment centers in the Netherlands (Academic Medical Center Amsterdam, Erasmus University Medical Center Rotterdam and Leiden University Medical Center) or a member of the Dutch Hemophilia Patient Society Young Adult committee (DHPSYA; which includes patients with different congenital bleeding disorders meeting the inclusion criteria).

### Procedure

After approval by the Medical Ethics Committees of the Academic Medical Centre, invitational letters, including login codes for online questionnaires, were sent out between May and October 2015 to the eligible patients of the medical centers and to members of the DHPSYA. To recruit additional respondents, the DHPSYA emailed their members, and posted an online call for participation on their website and Facebook page. Informed consent was obtained from all participants.

### Measures

#### Sociodemographics and medical characteristics

Sociodemographics of the participants were assessed with questions from the Course of Life Questionnaire (CoLQ) [18, 19], regarding age, gender, ethnicity, education, employment, and marital status. Education was divided into three categories according to the classification of Statistics Netherlands; low (primary education, lower vocational education, lower and middle general secondary education), middle (middle vocational education, higher secondary education, pre-university education), high (higher vocational education, university). In addition, the respondents were asked medical questions regarding the type of disease, severity [severe hemophilia: <1% clotting factor present in blood, non-severe hemophilia (1–40% clotting factor)], treatment, and number of bleedings that occurred over the past 6 months that required treatment.

#### Pediatric Quality of Life Inventory (PedsQL 4.0) generic core scales young adult version

The Dutch version of the Pediatric Quality of Life Inventory generic core scales young adult version (PedsQL_YA) was used [20, 21]. The PedsQL_YA is a generic self-report HRQOL instrument developed for YA aged 18–30 years and contains 23 items in four scales; physical health (eight items), emotional functioning (five items), social functioning (five items), and work/school functioning (five items). A psychosocial health scale score and a total scale score can be computed. Answers are divided over a 5-point Likert scale, consisting of the options ‘never’ (0) to ‘almost always’ (4). Each answer is reversed scored and rescaled to a 0–100 scale. Higher scores on the PedsQL_YA indicate better reported HRQOL. The validity and reliability of the PedsQL scales are good [21]. The internal consistencies (Cronbach’s alpha) in our sample ranged from .72 (social functioning) to .90 (total scale). A norm group of 649 YA peers from the Dutch population (18–30 years), healthy and with chronic illnesses, was available [21].

#### Course of life questionnaire (CoLQ)

The Course of life questionnaire (CoLQ) was used to assess the psychosocial developmental trajectory retrospectively (course of life; CoL) [18, 19]. The CoLQ has 74 items in five scales, concerning behaviors that are characteristic of certain age stages, developmental tasks, and the limitations patients might encounter when growing up with a chronic illness. We used three scales of the CoLQ that covered psychosocial development: development of autonomy (six items, score 6–12), social development (12 items, score 12–24), and psychosexual development (four items, score 6–12). A higher score on the scales indicates the accomplishment of more developmental milestones and therefore a more favorable course of life [19]. The validity and the test–retest reliability of the CoLQ scales are good [18, 19]. The internal consistencies (Cronbach’s alpha) in our sample ranged from 0.48 (development of autonomy) to 0.77 (psychosexual development). A recently updated norm group of 655 YA peers from the general Dutch population (18–30 years) was available.

#### Rosenberg self-esteem scale (RSES)

The Rosenberg self-esteem scale (RSES) was used to assess self-esteem [22]. The RSES measures the self-acceptance aspect of self-esteem or the overall sense of being capable, worthwhile, and competent. The RSES is a self-administered questionnaire with 10-items on a 4-point scale, ranging from one (strongly agree) to four (strongly disagree), with possible scores ranging from 10 to 40 [22]. A higher score indicates a higher self-esteem. The internal consistency (Cronbach’s alpha) in our sample was 0.80. A norm group of 1002 YA peers (18–30 years) was available [23].

### Data analysis

The Statistical Package for Social Sciences (SPSS) version 23.0 was used for all statistical analyses [24]. First, preparatory analyses were performed: calculation of internal consistencies (Cronbach’s alpha), computing of scale scores, and the distributions of scale scores were considered.

Next, descriptive analyses were performed to describe the sample. Demographic characteristics of our sample and the norm groups or peers were compared (age, gender, ethnicity, education, employment, and marital status) using *χ* ^2^-tests for categorical data and *t* tests for continuous data.

Differences on the PedsQL scale scores, CoLQ scales, and RSES between the patients with bleeding disorders and their peers were analyzed with Mann–Whitney *U* tests. Differences on the scale items of the CoLQ, which represent the achievement of the individual milestones, were analyzed with *χ* ^2^-tests. To adjust for multiple testing, we used a Bonferroni correction for the PedsQL YA (0.05/6 = 0.008) and CoLQ scale scores (0.05/3 = 0.017) and as well as for the individual milestones (autonomy: 0.05/6 = 0.008, social development: 0.05/12 = 0.004, and psychosexual development: 0.05/4 = 0.013).

Since hemophilia affects primarily men, the sample of YA with bleeding disorders had a significantly higher percentage (*p* < 0.01, 82%) of men than the peer groups (49%). In addition, bleeding disorders have a different impact on men and women, and therefore we analyzed the outcomes for gender separately. Since bleeding disorders other than hemophilia have a less clear distinction in severity, differences between YA men with severe and non-severe hemophilia were analyzed only, using Mann–Whitney *U* tests.

For the results of the Mann–Whitney *U* tests, effect sizes (*r*) were calculated by dividing the Z-scores by the square root of the sample size. Effect sizes up to 0.1, 0.3, and 0.5 were considered to be small, moderate, and large, respectively [25].

## Results

### Sociodemographic and medical characteristics

In total, 95 YA completed the online questionnaires. From the Academic Medical Center Amsterdam 34 YA participated (46% response rate), from the Erasmus University Medical Center Rotterdam 37 YA participated (46% response rate), from Leiden University Medical Center 10 YA participated (33% response rate), from the DHPSYA 10 YA participated (83% response rate), and the online call yielded another 3 YA (response rate unknown). The mean age of the 78 men (82.1%) was 24.7 years (SD 3.5) and of the 17 women (17.9%) 25.1 years (SD 3.8). Table 1 represents the sociodemographic and medical characteristics of the study population. The YA men in our sample were more often not employed in a paid job (*p* < 0.05) than peers in the PedsQL and CoLQ norm populations. Age, educational level, ethnicity, and marital status did not differ from these norm populations. Age did not differ from the RSES norm population.

**Table 1:** Sociodemographic and medical characteristics of young adults with bleeding disorders

	Men			Women		
	*N*	M	SD	*N*	M	SD
Age	78	24.7	3.5	17	25.1	3.8
Ethnicity (Dutch)	69	88.5 (%)		16	94.1 (%)	
Education^†^						
High	19	24.4 (%)		7	41.2 (%)	
Middle	43	55.1 (%)		6	35.3 (%)	
Low	16	20.5 (%)		4	23.5 (%)	
Employment (paid job)	45	57.7 (%)		12	70.6 (%)	
Marital status (married/living together)	21	26.9 (%)		6	35.3 (%)	
Type of bleeding disorder						
Hemophilia A	55	70.5 (%)		1	5.9 (%)	
Hemophilia B	15	19.2 (%)		0	0 (%)	
Von Willebrand type 2/3	6	7.7 (%)		10	58.8 (%)	
Other congenital bleeding disorder	2	2.6 (%)		6	35.3 (%)	
Type of treatment bleeding disorder						
Prophylaxis	37	47.5 (%)		1	5.9 (%)	
On demand—in case of bleed	41	52.6 (%)		16	94.1 (%)	
Severity of hemophilia						
Non-severe (>1%)	36	51.4 (%)		1	5.9 (%)	
Severe (<1%)	34	48.6 (%)		0	0 (%)	

	Men		Women	
	Median	Range	Median	Range
Number of bleeds past 6 months requiring treatment	1.00	0–20.00	0	0–20.00

*N* number, *M* mean, *SD* standard deviation

^†^ *Highest level completed: Low*: primary education, lower vocational education, lower and middle general secondary education; *Middle*: middle vocational education, higher secondary education, pre-university education; *High*: higher vocational education, university

### HRQOL

YA men with bleeding disorders reported significantly lower HRQOL on the total scale, physical health scale, and school/work functioning scale in comparison to healthy men on the PedsQL_YA, with small effect sizes (see Table 2). There were no differences found between HRQOL of YA with bleeding disorders and HRQOL of peers with chronic illnesses. YA men with severe hemophilia (median 81.25, mean 78.86, SD 19.39) reported lower physical functioning than men with non-severe hemophilia (median 93.75, mean 93.06, SD 7.21, *p* < 0.01, *r* = 0.45) on the PedsQL_YA. HRQOL scores did not differ on the other PedsQL_YA scales between severity groups (data not shown).

The HRQOL of YA women with bleeding disorders did not differ significantly from their healthy or chronically ill peers.

**Table 2:** PedsQL scale scores and differences between young adults with bleeding disorders and the PedsQL norm groups

PedsQL (sub)scale	Bleeding disorders			Healthy peers			Chronic ill peers^a^			Bleeding disorders versus healthy		Bleeding disorders versus chronic ill	
	*N*	Median (range)	Mean (SD)	*N*	Median (range)	Mean (SD)	*N*	Median (range)	Mean (SD)	*p* Value	Effect size *r*	*p* Value	Effect size *r*
Men													
Total score	78	84.78 (60–100)	83.65 (11.85)	267	90.22 (33–100)	87.51 (11.88)	50	86.96 (37–100)	82.48 (13.30)	0.00*	0.14	0.73	0.05
Physical health	78	90.63 (37–100)	86.18 (15.99)	267	96.88 (0–100)	91.50 (12.82)	50	93.75 (37–100)	87.13 (14.69)	0.00*	0.16	0.89	0.03
Emotional functioning	78	80.00 (25–100)	81.09 (16.65)	267	85.00 (15–100)	82.36 (17.25)	50	82.50 (20–100)	78.80 (18.17)	0.44	0.03	0.55	0.07
Social functioning	78	90.00 (45–100)	86.41 (13.79)	267	95.00 (20–100)	89.46 (13.28)	50	90.00 (35–100)	84.20 (17.45)	0.06	0.10	0.59	0.07
School/work functioning	78	80.00 (35–100)	79.42 (15.46)	267	85.00 (20–100)	84.33 (14.81)	50	80.00 (30–100)	77.00 (16.48)	0.01*	0.14	0.57	0.08
Psychosocial health	78	81.67 (47–100)	82.31 (13.03)	267	88.33 (28–100)	85.38 (13.37)	50	83.33 (37–100)	80.00 (14.44)	0.03	0.10	0.43	0.08
Women													
Total score	17	80.43 (49–100)	78.64 (13.15)	245	84.78 (52–100)	84.10 (10.70)	87	75.00 (24–100)	73.30 (16.40)	0.09	0.12	0.22	0.12
Physical health	17	87.50 (38–100)	80.88 (16.75)	245	90.63 (16–100)	87.53 (12.89)	87	78.13 (9–100)	72.59 (23.57)	0.07	0.12	0.28	0.14
Emotional functioning	17	65.00 (45–100)	69.41 (16.29)	245	75.00 (25–100)	74.39 (17.05)	87	70.00 (10–100)	68.56 (18.33)	0.17	0.07	0.74	0.02
Social functioning	17	90.00 (50–100)	86.18 (86.18)	245	90.00 (40–100)	88.04 (13.31)	87	80.00 (20–100)	79.43 (16.77)	0.73	0.03	0.08	0.15
School/work functioning	17	80.00 (45–100)	76.76 (16.48)	245	85.00 (20–100)	84.39 (13.97)	87	75.00 (20–100)	73.05 (18.70)	0.05	0.13	0.46	0.08
Psychosocial health	17	78.33 (55–100)	77.45 (12.32)	245	83.33 (52–100)	82.27 (11.93)	87	76.67 (25–100)	73.68 (15.53)	0.12	0.10	0.37	0.09

Higher scores indicate better HRQOL

*N* number, *M* mean, *SD* standard deviation

* Difference at *p* < 0.008, adjusted for multiple testing

^a^ Most common conditions chronically ill sample were: asthma (34.3%), psychiatry (10.9%), gastro enterology (10.2%), and skin disease (5.8%)

### Developmental milestones

Regarding YA men with bleeding disorders, no differences were found compared with peers on autonomy development scale level. When looking at the single items (milestones) in the scale, the sample of YA men with bleeding disorders showed a higher score in ‘paid chores, elementary school,’ but a delay in ‘paid jobs, during middle and/or high school.’ On the social development scale, no differences were found on a scale level between YA men with bleeding disorders and their peers, nor on item level. YA men with bleeding disorders scored significantly higher than their peers on the psychosexual development scale, indicating that they achieved more milestones (see Table 3). When looking at the single items (milestones) in the scale, the sample of YA men with bleeding disorders reported a higher score on ‘first time sexual intercourse <18 years’ than their peers (see Table 5 in Appendix). With regard to severity of hemophilia in men, no differences were found in the achievement of developmental milestones on the scales or at item level (data not shown).

**Table 3:** CoLQ scale scores and differences between young adults with bleeding disorders and the CoLQ norm group

CoLQ scale	Bleeding disorders			Norm group			Bleeding disorders versus norm group	
	*N*	Median (range)	Mean (SD)	*N*	Median (range)	Mean (SD)	*p* Value	Effect size *r*
Men								
Autonomy development	78	9.00 (6–12)	8.69 (1.61)	321	9.00 (6–12)	8.95 (1.44)	0.21	0.07
Social development	78	21.00 (14–24)	20.42 (3.05)	321	21.00 (12–24)	20.12 (2.89)	0.35	0.04
Psychosexual development	78	8.00 (4–8)	7.14 (1.20)	321	7.00 (4–8)	6.64 (1.38)	0.00*	0.15
Women								
Autonomy development	17	9.00 (7–11)	8.82 (1.38)	334	9.00 (6–12)	9.22 (1.46)	0.29	0.06
Social development	17	22.00 (16–24)	20.76 (2.59)	334	21.00 (12–24)	20.33 (2.59)	0.51	0.04
Psychosexual development	17	7.00 (4–8)	6.76 (1.39)	334	8.00 (4–8)	6.94 (1.35)	0.57	0.03

Higher scores indicate better CoL

*N* number, *M* mean, *SD* standard deviation

* Difference at *p* < 0.017, adjusted for multiple testing

Regarding YA women with bleeding disorders, no differences were found compared with peers on autonomy development scale level. However, when looking at the single items (milestones) in the scale, the sample of YA women with bleeding disorders showed a delay in ‘paid jobs, during middle and/or high school.’ No differences were found between YA women with bleeding disorders and their peers on the social development and psychosexual development scales, nor at item level, of the CoLQ (see Table 3 and Appendix Table 6).

### Self-esteem

YA men with bleeding disorders reported significantly lower self-esteem than their peers (Table 4), with a small effect size. No differences were found between hemophilia severity groups (data not shown). Self-esteem of YA women with bleeding disorders did not differ significantly from their peers.

**Table 4:** RSES scores and differences between young adults with bleeding disorders and the RSES norm group

	Self-Esteem	Bleeding disorders			Norm group			Bleeding disorders vs norm	
		*N*	Median (range)	Mean (SD)	*N*	Median (range)	Mean (SD)	*p* Value	Effect size *r*
Men^a^	Total scale	77	33.00 (18–37)	31.78 (4.34)	443	33.00 (14–40)	33.36 (4.32)	0.01*	0.13
Women	Total scale	17	29.00 (22–37)	30.41 (3.86)	559	31.00 (16–40)	31.32 (4.65)	0.30	0.03

Higher scores indicate higher self-esteem

*N* number, *M* mean, *SD* standard deviation

* Difference at *p* < 0.05

^a^ One YA man did not complete the RSES

## Discussion

This study demonstrates that YA men with bleeding disorders show slight impairments in total HRQOL, physical functioning, school/work functioning (PedsQL_YA), and self-esteem (RSES), in comparison to their (healthy, sex-matched) peers. No differences were found on the CoLQ scales (autonomy and social development), except for psychosexual development.

The YA (aged 18–30 years) in our patient population have been born just after the hepatitis and HIV crises [2]. The current generation of YA with bleeding disorder is therefore expected to grow up with less problems than previous generations. However, our results show that growing up with a bleeding disorder these days still can have a negative impact on daily life of YA, which is in line with research on YA growing up with a chronic illness [21]. Despite adequate treatment, YA men with bleeding disorders still experience moderately impaired physical functioning compared to their healthy peers, especially those with severe hemophilia. Although bleeds occur less frequent than in previous generations, joint bleeds cause cumulative and irreversible damage to joints, with arthropathy and limited physical functioning as a result when boys grow up to be YA.

The finding that YA men with bleeding disorders are still facing difficulties in daily life underlines the findings from the focus groups [17]. This study covers three of the important themes from the focus groups. In line with the qualitative results, we found difficulties with school/work functioning and self-esteem. However, not all results based on the outcomes of the questionnaires are in concordance with the findings from the focus groups. For example, during the focus groups, autonomy development (e.g., gaining independence from parents) was mentioned as challenging, which was not confirmed in this study.

The current findings regarding school/work functioning is not only in line with our former focus group study, but also with other studies. For example, previous research in 2001 in the Netherlands demonstrated that men with severe hemophilia participated less in full-time work compared with the general population [26]. In addition, on the sociodemographic characteristics of our sample, we found a significantly lower rate of paid jobs in YA men with bleeding disorders compared to peers, which has also been found in studies in the US [27, 28]. Of the YA men in our sample, 50% indicated they were still in college, while their peers were already working. An explanation could be that YA men with bleeding disorders are more likely to apply for ‘white collar’ jobs, which are often more suitable jobs for hemophilia patients, since the risk of bleeding due to physical activities in this type of work is lower [26]. In this case, patients with bleeding disorders are more likely to follow full-time education over a longer period of time.

The problems related to school or professional functioning are also interesting in relation to the finding that young adult men with bleeding disorders report a somewhat lower self-esteem than their peers. One can imagine that when a YA feels less successful in a professional sense, or when a YA is not able to pursue professional goals due to physical limitations, this could lead to a decreased self-esteem. Work experience during adolescence, which has shown to be less frequent in adolescents with bleeding disorders, is not only an excellent way to discover skills and interests, but also to experience negative feelings related to limitations of physical capacities [29]. Finding an education or job that fits with some of the physical restrictions that YA with bleeding disorders encounter is important, and guidance and support is desirable, for example, by a social worker or professional coach and strengthen work-related psychosocial skills [30–33]. As an example, Emma at Work, a job mediation agency for YA with chronic diseases at the Academic Medical Center in Amsterdam, can be a useful service [33].

YA men reported to have achieved slightly more psychosexual developmental milestones than their peers (‘first time intercourse <18 years’). Although this result is in contradiction to our expectations, this finding suggests that YA men growing up with bleeding disorders do not feel limited by their condition, with regard to discovering psychosexual relations during adolescence. However, the CoLQ used in this study measures retrospectively. This does not imply that sexual functioning at present is not hindered by their bleeding disorder or physical impairments, which may have decreased over the years due to bleeds. For example, (fear of) pain, or fear of bleeding during intercourse, may affect sexual functioning. Furthermore, arthropathy in joints may place limitations on sexual functioning, while women with bleeding disorders may experience impaired sexuality due to long lasting heavy menstruation [34, 35]. It would be interesting to explore current sexual functioning more for this population, especially since patients seem to be keen to talk about this subject with their healthcare providers [36]. Psychosexual development and functioning of YA receives very little attention in clinical practice and in literature, and it is important to study this aspect more in depth in the future [35–37].

YA women with bleeding disorders do not show any impairments in HRQOL, psychosocial development on scale level, and self-esteem in comparison to their peers. Only on the autonomy scale of the CoLQ, women with bleeding disorders scored lower on the item ‘paid jobs, middle and/or high school.’ We assume that because women experience less joint bleeds and consequences from their condition compared to men, this results in less impaired functioning in daily life. Research has shown that women with bleeding disorders are impacted majorly by menstrual disorders compared with their peers, especially during teenage years [38]. Unfortunately, the questionnaires used in this study do not cover questions regarding menorrhagia, which would be interesting to include in the future when studying women with bleeding disorders. Still, we wanted to include this group of young adult women, albeit so small, because research on women with bleeding disorders is scarce. The small sample size causes warranty however in generalization of results.

Some limitations of this study should be taken into account. First, the internal consistency of the autonomy scale was low. However, we looked at individual items on the CoLQ scales to overcome this issue. In addition, these internal consistencies are not exceptional and are in concordance with other studies using the CoLQ [39, 40]. Secondly, we did not have all sociodemographic and medical information of non-respondents. Thirdly, the exact response rate of our study is unknown, due to the fact that we recruited additional participants through on online call. We decided to add this call, because recruitment was quite difficult, possibly due to the time consuming nature of completing the questionnaires. Therefore, we do not know whether the results are representative for YA with bleeding disorders and we could be dealing with an under- or overestimation of the problems of this group YA. Also, effect sizes found in our study are quite small. This should be kept in mind when interpreting and generalizing the results. In addition, it would be interesting to collect longitudinal data during the transitional phase to identify factors that may influence HRQOL and psychosocial adaptation in YA with bleeding disorders, especially because the problems seem to increase from childhood to adulthood [41]. Adding a disease-specific HRQOL questionnaire, which is usually more sensitive for changes over time (e.g., Hemophilia Well-Being Index [42] or HAEMO-QoL-A [43]), can be valuable in collecting longitudinal HRQOL data [44, 45].

In conclusion, this study demonstrates that YA men with bleeding disorders show some impairments in physical functioning, school/work functioning, and self-esteem. Although these YA are functioning quite well, systematic monitoring of HRQOL and psychosocial functioning over time in YA with bleeding disorders in daily clinical practice is important since possible influencing psychosocial factors can change over time. This way, problems can be identified early, especially with regard to their physical and professional/school functioning, in order to optimize their well-being and adaptation to society in the process of transition to adulthood.

## Appendix

See Tables 5 and 6.

**Table 5:** CoLQ item scores and differences between young adult men with bleeding disorders and the CoLQ norm group

Milestones	Men with bleeding disorders (*n* = 78)		Norm group (*n* = 321)		*p*
	*N*	%	*N*	%	
Autonomy development					
Regular chores/tasks in your family, elementary school					
Yes	31	39.7	121	37.7	0.80
No	47	60.3	200	62.3	
Paid chores, elementary school					
Yes	33	42.3	78	24.3	**0.00***
No	45	57.7	243	75.7	
Regular chores/tasks in your family, middle and/or high school					
Yes	42	53.8	160	49.8	0.53
No	36	46.2	161	50.2	
Paid jobs, middle and/or high school					
At the age of 18 or younger	29	37.2	275	85.7	**0.00***
At the age of 19 or older/never	49	62.8	46	14.3	
For the first time vacation without adults					
At the age of 17 or younger	31	39.7	132	41.1	0.90
At the age of 18 or older/never	47	60.3	189	58.9	
Leaving your parents home					
Not living with your parents	44	56.4	181	56.4	1.00
Still living with your parents	34	43.6	140	43.6	
Social development					
At least one year of membership in a sports club/competitive sports, elementary school					
Yes	58	74.4	260	81.0	0.21
No	20	25.6	61	19.0	
Number of friends in kindergarten through third grade, elementary school					
4 or more	44	56.4	194	60.4	0.52
Less than 4	34	43.6	127	39.6	
Number of friends in fourth–sixth grade, elementary school					
4 or more	51	65.4	198	61.7	0.60
Less than 4	27	34.6	123	38.3	
Best friend, elementary school					
Yes	62	79.5	217	67.6	0.04
No	16	20.5	104	32.4	
Most of the time playing with…, elementary school					
Friends	65	83.3	268	83.5	1.00
Brothers and/or sisters, parents, on your own	13	16.7	53	16.5	
At least one year of membership in a sports club/competitive sports, middle and/or high school					
Yes	46	59.0	206	64.2	0.43
No	32	41.0	115	35.8	
Number of friends, middle and/or high school					
4 or more	53	67.9	200	62.3	0.43
less than 4	25	32.1	121	37.7	
Best friend, middle and/or high school					
Yes	52	66.7	186	57.9	0.20
No	26	33.3	135	42.1	
Belonging to a group of friends, middle and/or high school					
Yes	62	79.5	246	76.6	0.65
No	16	20.5	75	23.4	
Leisure time, mainly with …, middle and/or high school					
Friends	65	83.3	257	80.1	0.63
Brothers and/or sisters, parents, on your own	13	16.7	64	19.9	
Going out to a bar or disco, middle and/or high school					
Sometimes/often	60	76.9	236	73.5	0.57
Never	18	23.1	85	26.5	
At least one year of membership in a sports club/competitive sports, after middle and/or high school					
Yes	39	50.0	138	43.0	0.31
No	39	50.0	183	57.0	
Psychosexual development					
First girlfriend/boyfriend					
At the age of ≤17 years	54	69.2	190	59.2	0.12
At the age of ≥18 years	24	30.8	131	40.8	
For the first time falling in love					
At the age of ≤18 years	74	94.9	274	85.4	0.02
At the age of ≥19 years	4	5.1	47	14.6	
For the first time sexual intimacy					
At the age of ≤18 years	62	79.5	221	68.6	0.07
At the age of ≥19 years	16	20.5	100	31.2	
For the first time sexual intercourse					
At the age of ≤18 years	55	70.5	164	51.1	**0.00*****
At the age of ≥19 years	23	29.5	157	48.9	

** p* < 0.008 based on *χ* ^2^ adjusted for multiple testing (autonomy development)

*** p* < 0.004 based on *χ* ^2^ adjusted for multiple testing (social development)

*** *p* < 0.013 based on *χ* ^2^ adjusted for multiple testing (psychosexual development)

**Table 6:** CoLQ item scores and differences between YA women with bleeding disorders and the CoLQ norm group

Milestones	Women with bleeding disorders (*n* = 17)		Norm group (*n* = 334)		*p*
	*N*	%	*N*	%	
Autonomy development					
Regular chores/tasks in your family, elementary school					
Yes	6	35.3	138	41.3	0.80
No	11	64.7	196	58.7	
Paid chores, elementary school					
Yes	6	35.3	78	23.4	0.25
No	11	64.7	256	76.6	
Regular chores/tasks in your family, middle and/or high school					
Yes	8	47.1	182	54.5	0.62
No	9	52.9	152	45.5	
Paid jobs, middle and/or high school					
At the age of 18 or younger	9	52.9	282	84.4	**0.00***
At the age of 19 or older/never	8	47.1	52	15.6	
For the first time vacation without adults					
At the age of 17 or younger	7	41.2	147	44.0	1.00
At the age of 18 or older/never	10	58.8	187	56.0	
Leaving your parents home					
Not living with your parents	12	70.6	250	74.9	0.78
Still living with your parents	5	29.4	84	25.1	
Social development					
At least one year of membership in a sports club/competitive sports, elementary school					
Yes	14	82.4	266	79.6	1.00
No	3	17.6	68	20.4	
Number of friends in kindergarten through third grade, elementary school					
4 or more	13	76.5	199	59.6	0.21
Less than 4	4	23.5	135	40.4	
Number of friends in fourth-sixth grade, elementary school					
4 or more	12	70.6	197	59.0	0.45
Less than 4	5	29.4	137	41.0	
Best friend, elementary school					
Yes	14	82.4	254	76.0	0.77
No	3	17.6	80	24.0	
Most of the time playing with….., elementary school					
Friends	16	94.1	282	84.4	0.49
Brothers and/or sisters, parents, on your own	1	5.9	52	15.6	
At least one year of membership in a sports club/competitive sports, middle and/or high school					
Yes	10	58.8	203	60.8	1.00
No	7	41.2	131	39.2	
Number of friends, middle and/or high school					
4 or more	11	64.7	206	61.7	1.00
Less than 4	6	35.3	128	38.3	
Best friend, middle and/or high school					
Yes	16	94.1	247	74.0	0.08
No	1	5.9	87	26.0	
Belonging to a group of friends, middle and/or high school					
Yes	12	70.6	269	80.5	0.35
No	5	29.4	65	19.5	
Leisure time, mainly with….., middle and/or high school					
Friends	15	88.2	290	86.8	1.00
Brothers and/or sisters, parents, on your own	2	11.8	44	13.2	
Going out to a bar or disco, middle and/or high school					
Sometimes/often	9	52.9	249	74.6	0.09
Never	8	47.1	85	25.4	
At least one year of membership in a sports club/competitive sports, after middle and/or high school					
Yes	7	41.2	120	35.9	0.80
No	10	58.8	214	64.1	
Psychosexual development					
First girlfriend/boyfriend					
At the age of ≤ 17 years	10	58.8	225	67.4	0.44
At the age of ≥ 18 years	7	41.2	109	32.6	
For the first time falling in love					
At the age of ≤ 18 years	14	82.4	293	87.7	0.46
At the age of ≥ 19 years	3	17.6	41	12.3	
For the first time sexual intimacy					
At the age of ≤ 18 years	15	88.2	253	75.7	0.38
At the age of ≥ 19 years	2	11.8	81	24.3	
For the first time sexual intercourse					
At the age of ≤ 18 years	8	47.1	212	63.5	0.20
At the age of ≥ 19 years	9	52.9	122	36.5	

* *p* < 0.008 based on *χ* ^2^ adjusted for multiple testing (autonomy development)

** *p* < 0.004 based on *χ* ^2^ adjusted for multiple testing (social development)

*** *p* < 0.013 based on *χ* ^2^ adjusted for multiple testing (psychosexual development)

## Abbreviations

HRQOL: Health-Related Quality of Life
YA: Young adults
PedsQL_YA: Pediatric Quality of Life Inventory Young Adult Version
CoLQ: Course of Life Questionnaire
RSES: Rosenberg Self-Esteem Scale
DHPSYA: Dutch Hemophilia Patient Society Young Adult committee
